# The extraordinary variation of the organellar genomes of the Aneura pinguis revealed advanced cryptic speciation of the early land plants

**DOI:** 10.1038/s41598-017-10434-7

**Published:** 2017-08-29

**Authors:** Kamil Myszczyński, Alina Bączkiewicz, Katarzyna Buczkowska, Monika Ślipiko, Monika Szczecińska, Jakub Sawicki

**Affiliations:** 1 Department of Botany and Nature Protection, University of Warmia and Mazury, Plac Łódzki 1, 10-727 Olsztyn, Poland; 2 Department of Biology, Institute of Experimental Biology, Adam Mickiewicz University in Poznań, Umultowska 89, 61-614 Poznań, Poland

## Abstract

*Aneura pinguis* is known as a species complex with several morphologically indiscernible species, which are often reproductively isolated from each other and show distinguishable genetic differences. Genetic dissimilarity of cryptic species may be detected by genomes comparison. This study presents the first complete sequences of chloroplast and mitochondrial genomes of six cryptic species of *A. pinguis* complex: *A. pinguis* A, B, C, E, F, J. These genomes have been compared to each other in order to reconstruct phylogenetic relationships and to gain better understanding of the evolutionary process of cryptic speciation in this complex. The chloroplast genome with the nucleotide diversity 0.05111 and 1537 indels is by far more variable than mitogenome with π value 0.00233 and number of indels 1526. Tests of selection evidenced that on about 36% of chloroplast genes and on 10% of mitochondrial genes of *A. pinguis* acts positive selection. It suggests an advanced speciation of species. The phylogenetic analyses based on genomes show that *A. pinguis* is differentiated and forms three distinct clades. Moreover, on the cpDNA trees, *Aneura mirabilis* is nested among the cryptic species of *A. pinguis*. This indicates that the *A. pinguis* cryptic species do not derive directly from one common ancestor.

## Introduction

Complexes of cryptic species are groups of related species that are virtually identical or morphologically very similar, with unclear morphological boundaries between them. On the other hand, cryptic species often are reproductively isolated from each other and show distinguishable genetic differences, as great as those observed in taxonomically distinct species, with clear morphological differences^1–3^. Complexes of cryptic species occur in many groups of organisms, for instance, in bryophytes^4^, which include three early diverging lineages of land plants – liverworts, mosses, and hornworts. In the bryophyte group, cryptic speciation was detected in many seemingly widespread species such as, e.g.: *Mielichhoferia elongata* (Hoppe & Hornsch) Ness & Hornsch^2^, *Hamatocaulis vernicosus* (Mitt.) Hedenäs^5^, *Platyh* ***y*** *pnidum riparioides* (Hedw.) Dixon^6^, in the genera *Orthotrichum* (Hedw.) Sawicki^7^ and *Scleropodium* (Bruch & Schimp)^8^, *Targonia lorbeeriana* ^9^, in the genus of *Herbertus* ^10^, *Ptilidium ciliare* (L.) Hampe^11^, *Porella platyphylla* (L.) Pfeiff.^12^, *Frulania tamarisci* (L.) Dumort^13^ and in *Aneura pinguis* (L.) Dumort^3, 14^.

Genetic differences between species in the complexes of cryptic species may be detected by comparing their genomes. Next-generation sequencing enables sequencing complete genomes instead of a few genes and detecting the position of genes in the genome and differences between the nucleotides in genes. Also, by comparing mitochondrial or chloroplast genomes of cryptic species, it is possible to observe which genes are important for the speciation and evolution of bryophytes and which genes are subject to selection. However, only a few complete mitochondrial^15–19^ and chloroplast^20–22^ genomes have been published for bryophytes. Comparative analyses of mtDNA and cpDNA genomes in this group showed that bryophytes possess similar gene order and content^16–18^. Moreover, it is found that organelle genomes in bryophytes evolve slowly and show similarity to organelle genomes of charophyte algae apart from some features indicating dynamics, such as: moderate increase in genome size, large-scale intron gains and occurrence of RNA editing^23, 24^. In contrast, bryophyte genomes are dramatically different from the genomes of angiosperm plants that exhibit dynamic evolution of many features^17, 25, 26^.

*A. pinguis* is a thalloid liverwort with a simple morphological and anatomical structure and broad holarctic distribution – it ranges from Europe, Asia, Australia and New Zealand to North America and Mexico^27, 28^. It is common from lowlands up to the high mountain zone and grows in various habitats: on calcareous rocks, basic humus, peat bogs, wet sand on lake shores, and fallen decorticated logs^29^. It is now known as a species complex with several morphologically indistinguishable species temporarily named: *A. pinguis* species from A to L^3, 14, 30^. Genetic differences between them are clear and species may be distinguished by isozyme markers, ISSR markers and DNA barcodes^3, 14^.

In this study, is presented the first complete sequences of chloroplast and mitochondrial genomes of six cryptic species of *A. pinguis* complex: *A. pinguis* A, B, C, E, F and J. Additionally, these genomes have been compared to each other in order to reconstruct phylogenetic relationships between the studied species and to gain a better understanding of the evolutionary process of cryptic speciation in this complex.

## Materials and Methods

### Organellar genome sequencing and annotation

Total genomic DNA of six *Aneura pinguis* cryptic species was extracted from *in vitro* culture using the Zymo Plant/Seed DNA kit (Zymo, Hilden, Germany). Fresh thalli were ground with silica beads in a MiniBead-Beater tissue disruptor for 50 s, and they were subsequently processed using the manufacturer’s protocols. DNA quantity was estimated with the use of the Qubit fluorometer system (Invitrogen, Carlsbad, NM, USA) and the Quant-IT ds-DNA BR Assay kit (Invitrogen).

A genomic library for MiSeq sequencing was developed with the use of the Nextera XT Kit. DNA in the amount of 1 ng was used in the procedure described in the Nextera XT protocol (Illumina, San Diego, CA, USA). The number and accuracy of libraries was verified with the use of primers whose sequences are given in the Sequencing Library qPCR Quantification Guide (Illumina). PCR reactions were performed in 20 µL of a reaction mixture containing 3 µL of library genomes, 1.0 µM of each primer, 1.5 mM MgCl_2_, 200 µL M dNTP (dATP, dGTP, dCTP, dTTP), 1 × PCR buffer and 1 U OpenExTaq polymerase (Open Exome, Warsaw, Poland). PCR reactions were performed under the following thermal conditions: (1) initial denaturation, 5 min at a temperature of 94 °C; (2) denaturation, 30 s at 94 °C; (3) annealing, 30 s at 52 °C; (4) elongation, 1 min at 72 °C and final elongation, 7 min at 72 °C. Stages 2–4 were repeated 34 times. The products of the PCR reaction were separated in the QIAxcel capillary electrophoresis system (Qiagen). Electrophoresis was performed using the QIAxcel High Resolution Kit with the 15–1000 bp alignment marker (Qiagen) and the 25–1000 bp DNA size marker (Qiagen). Standard OL500 settings were used as the electrophoresis program. Validated libraries were pooled according to the Nextera XT protocol. Genomic libraries were sequenced using the MiSeq 500v2 cartridge that supported the acquisition of 2 × 250 bp pair-end reads. The resulting reads were preliminarily assembled using the Velvet *de novo* assembler implemented in the Geneious R8 software^31^. First, the reads were cleaned by removing adaptor sequences and low quality reads with ambiguous sequences. Afterwards reads were assembled with k-mer length of 31 bp and minimum contig length of 100 bp along with other default parameters. Finally, produced contigs with similarity less than 70% to reference organellar genomes such as: *Aneura mirabilis, Pellia endiviifolia*, *Ptilidium pulcherrimum, Pleurozia purpurea, Treubia lacunosa* and *Marchantia polymorpha* were discarded from further analyses.

The flow chart for the *in silico* construction of the *A. pinguis* organellar genomes was identical to that presented in the previous study^32^.

The four junctions between single-copy segments and inverted repeats were confirmed using PCR-based product sequencing of the assembled genomes. Purified PCR products were sequenced in both directions using the ABI BigDye 3.1 Terminator Cycle Kit (Applied Biosystems, Foster City, CA, USA) and visualized with the ABI Prism 3130 Automated DNA Sequencer (Applied Biosystems). The sequences obtained with the Sanger method were aligned with the assembled genomes using the Geneious R8 assembly software^31^ to check for any differences.

The assembled organellar genomes were annotated with DOGMA^33^ and Geneious R8^31^. The specimens and sequencing details with GenBank numbers are given in Table 1.

**Table 1:** Sequencing details and specimens used in this study.

Sample ID	Cryptic species	POZW voucher number	Locality	Habitat	Geographic coordinates	Collection date and collector	Number of sequencing reads	Mitogenome length [bp]	Plastome length [bp]	Mitogenome accession number	Plastome accession number
T199-1	A	42826	S Poland, Tatra Mts, NE slope of Skupniów Upłaz Mt, slope above road	Humus over lime rocks	49.16°N 20.00°E	18.09.2012 AB, KB	19,196,548	121,105	165,867	KY702722	KY702721
A18-1	B	42793	Poland, Bieszczady Mts, valley of Beskidnik stream, sparsely used road along the steam	Clay soil	49.08°N 20.29°E	10.08.2012 KB	1,037,898	121,140	164,047	KU140427	KY242383
T178-1	C	42819	Poland, Tatra Mts, Sucha Woda Valley, slope above road along the stream	Humus	49.28°N 20.03°E	22.09.2012 AB, KB	4,075,008	120,927	164,989	NC026901	KY242382
T184-3	E	42815	S Poland, Tatra Mts, Valley of Biały Potok stream	Limestone rock in flowing water	49.16°N 19.57°E	16.09.2012 KB, AB	17,743,042	120,698	167,033	KY702723	KY702720
BS1-3	F	42818	Poland, Beskid Sądecki Mts, Kozłecki stream, moist slopes of the stream	Clay soil	49.26°N 20.26°E	3.09.2012 KB	3,793,416	120,898	164,984	KR817582	KY242384
JAP1-1	J	41053	Japan, Mount Lide, N slope	Humus	37.86°N 139.76°E	10.07.2005 MI	4,166,004	120,898	164,699	KZ242386	KY242385

Collectors: KB - Katarzyna Buczkowska, AB – Alina Bączkiewicz, MI – Misao Itouga.

### Polymorphism analyses

Chloroplast genomes of six cryptic species of *Aneura pinguis* were aligned using the MAFFT genome aligner^34^. Afterwards based on alignment of genomes polymorphism analysis was conducted separately for each coding sequence, intron and intergenic spacer. Every variation within aforementioned regions was identified as single nucleotide polymorphism (SNP) or insertion/deletion (indel) and counted using custom Python script. The nucleotide diversity values (π) were calculated for each coding and noncoding region using MEGA7 software^35^. Because the nucleotide diversity is based only on substitutions, number of indels and percent of polymorphic sites value^36^ are given for each region, however section ‘Results and Discussion’ refers only to the π value. Each SNP within coding sequence was tested if it affects the protein sequence and defined as synonymous or nonsynonymous SNP. Finally, variations were visualized using Circos software^37^ combined with Python script. The number of synonymous differences per synonymous site (dS), the number of nonsynonymous differences per nonsynonymous site (dN) and dN/dS ratio^38^ were also calculated for CDSs (Supplementary Table S2). For each gene with dN/dS ratio larger than 1, codon-based Z-test of selection was done. The above evolutionary analyses were conducted in MEGA7^35^. Using RAxML tree (100 bootstrap replications) and alignment of all liverworts species mentioned in Supplementary Table S1, we carried out branch-site statistical test for positive selection in HyPhy^39^ using the BUSTED algorithm^40^. The same polymorphism analyses strategy was used also for mitochondrial genomes. For comparison of mt and cp genomes and to examine if they are consistent with neutral evolution, the HKA test was done^41^. As outgroups mitogenome and plastome of *Marchantia polymorpha* were used. The HKA test was conducted in DnaSp v5 software^42^.

### Analysis of protein-coding genes

Protein-coding genes were predicted based on the closest known genomes of related species to the *Aneura pinguis* i.e. *Aneura mirabilis, Pellia endiviifolia*, *Ptilidium pulcherrimum* for plastome and *Pleurozia purpurea, Treubia lacunosa* and *Marchantia polymorpha* for mitogenome. Predictions were made using Geneious R8 software^31^ and BLAST tool^43^.

### Phylogenomics reconstruction

Bayesian inference and maximum likelihood methods were applied to infer phylogenetic relationships. To construct phylogenetic trees 19 mitochondrial and 17 plastid genomes of bryophytes were used. The specimens and taxonomy status details with GenBank numbers are given in Supplementary Table S1. *Marchantia polymorpha* genomes were used as root of generated phylogenetic trees. Bayesian inference phylogenetic analyses were estimated for 1,500,000 generations, sampling one out of every 100 generations of random trees using MrBayes v. 3.2.6^44^. Maximum likelihood analyses were conducted using RAxML^45^ where the stability of clades was assessed by 100 bootstrap replications. Beside whole genome sequences, phylogenetic reconstructions were also performed for partitioned dataset including protein-coding genes. The ML trees were calculated separately for synonymous and nonsynonymous sites using HyPhy software^39^.

Relative-rate test was conducted using Phyltest 2.0 software^46^. This two-cluster test^47^ examines the constancy of evolutionary rate for two lineages where an outgroup lineage is given. Because of input data limit only plastomic and mitogenomic protein-coding sequences were used for relative-rate test. Phyltest allows multiple sequences to be included in each of the lineages. In our dataset *A. pinguis* is represented by six different mitogenomic and plastomic protein-coding sequences. Poisson correction distance was used for relative-rate comparisons of protein sequences. Species selected for relative-rate test are shown in Table 2.

**Table 2:** Results of relative-rate test for *A. pinguis* and other species lineages.

Genome	Species	Outgroup	La-Lb	Evolutionary rate	Z
mt	*Pleurozia purpurea*	*Treubia lacunosa*	0.0810309	+	**27.9378**
mt	*Treubia lacunosa*	*Marchantia polymorpha*	0.0873281	+	**24.0475**
mt	*Tetraphis pellucida*	*Marchantia polymorpha*	−0.00486008	−	1.00038
mt	*Orthotrichum stellatum*	*Marchantia polymorpha*	−0.00653954	−	1.30667
mt	*Syntrichia filaris*	*Marchantia polymorpha*	−0.00451914	−	0.910135
**cp**	*Aneura mirabilis*	*Ptilidium pulcherrimum*	−0.0472961	−	−*
cp	*Ptilidium pulcherrimum*	*Pellia endiviifolia*	0.0926284	+	**25.0532**
cp	*Pellia endiviifolia*	*Marchantia polymorpha*	0.0833034	+	**21.8773**
cp	*Physcomitrella patens*	*Marchantia polymorpha*	0.033927	+	**7.9098**
cp	*Takakia lepidozioides*	*Marchantia polymorpha*	−0.0135805	−	**2.94158**
cp	*Nyholmiella obtusifolia*	*Marchantia polymorpha*	0.0351483	+	**8.18193**

La and Lb are the average numbers of substitutions per site (branch lengths) from the common ancestor (outgroup) of cluster A and B. “+” - faster evolutionary rate of protein-coding sequences of *A. pinguis* than that of the compared species, “−” - slower rate of evolution of *A. pinguis*. The bold font in Z column depict the statistically significant differences in evolutionary rates at the 5% level. “*” - two-tailed test cannot be computed probably because of substantial genetic differences between *A. mirabilis* and other bryophytes (besides *A. pinguis*).

## Results and Discussion

### Structure and polymorphism of chloroplast genome

**Figure 1:**
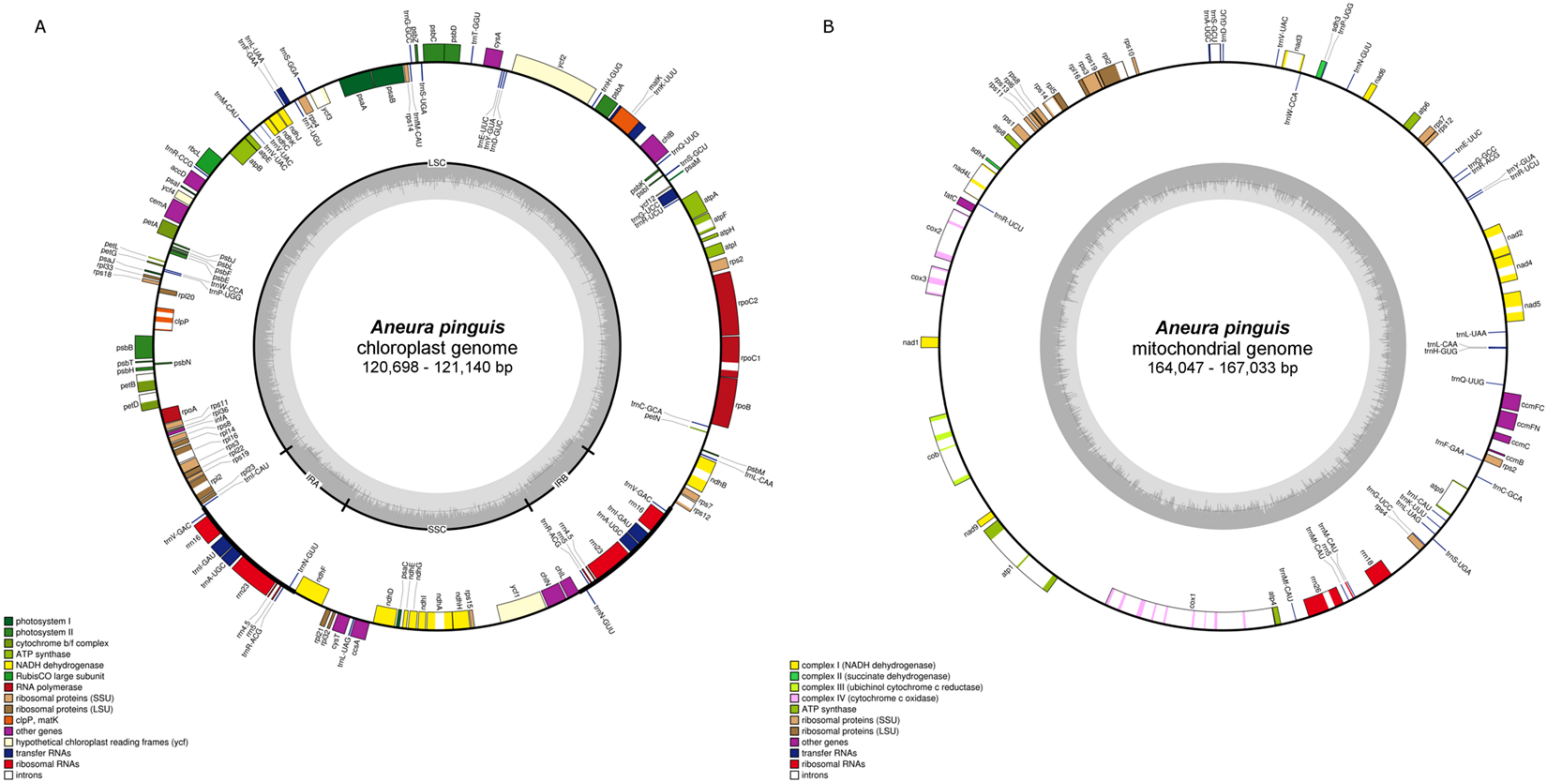
Genome maps of *Aneura pinguis* chloroplast (**A**) and mitochondrial (**B**) genomes. OGDraw genome maps^72^. Genes in the clockwise direction are on the inside of the map, and genes in the counterclockwise direction are on the outside of the map. Annotated genes are colored according to the functional categories shown in the legend. The inner circle visualizes GC content with midpoint line which indicates 50% GC content. Histogram presents GC content per 50 bp.

The chloroplast genomes of six cryptic species of *Aneura pinguis* length range from 120,698 to 121,140 bp and display the typical structure of most land plants, consisting of a pair of IRs (each of 8,575 bp) separated by LSC (83,632 bp) and SSC (20,145 bp) regions (Fig. 1A). Overall GC content of the cpDNA is 39.3%, which is similar to other known Jungermanniopsida class chloroplast genomes (33–41%)^48, 49^. The IR gene content is identical to that of the liverworts *Aneura mirabilis*, *Pellia endiviifolia* and *Ptilidium pulcherrimum*, including the *trn*V-GAC, *rrn*16, *trn*I-GAU, *trn*A-UGC, *rrn*23, *rrn*4.5, *rrn*5, *trn*R-ACG and *trn*N-GUU genes.

Of the 122 unique genes (i. e., including one copy of the inverted repeats) identified in *Aneura pinguis* plastome, 81 are protein coding genes, 5 genes of unknown function (*ycf* genes), 4 ribosomal RNAs and 32 transfer RNAs (Table 3). Ten genes such as: *atp*F, *ndh*A, *ndh*B, *pet*B, *pet*D, *rpl*2, *rpl*16, *rpo*C1, *rps*12 and *ycf*3 contain one intron and *clp*P contains two introns. The IRs and LSC gene content is identical to that of closely related *Aneura mirabilis*, the only member of Aneuraceae family with known chloroplast genome structure^49^. However, SSC gene content of *Aneura mirabilis* lacks such genes as *ndh*A, *ndh*G, *ndh*H and *ndh*I in comparison with *Aneura pinguis*. These chlororespiratory genes encode subunits of the NADH dehydrogenase complex in plant chloroplast genomes and play a role in photosynthesis^50^. The *ndh* genes are considered to be lacking or nonfunctional in heterotrophic plants^51^ and *Aneura mirabilis* is the only bryophyte species known to receive carbon from other source than photosynthesis^52^. Therefore *Aneura pinguis* plastome is 12,920 bp longer than that of *Aneura mirabilis* (108,007 bp).

**Table 3:** Genes contained within the chloroplast and mitochondrial genome of *Aneura pinguis*.

	Gene names	Type of gene
**cpDNA**	accD	Acetyl-CoA carboxylase
	atpA, atpB, atpE, atpF, atpH, atpI	ATP synthase
	clpP	Clp protease
	petA, petB, petD, petG, petL, petN	Cytochrome b/f complex
	ccsA	Cytochrome c biogenesis protein
	cemA	Envelope membrane protein
	ycf1, ycf2, ycf3, ycf4, ycf12	Hypothetical genes
	chlB, chlL, chlN	Light-independent protochlorophyllide oxidoreductase subunit
	matK	Maturase
	ndhA, ndhB, ndhC, ndhD, ndhE, ndhF, ndhG, ndhH, ndhI, ndhJ, ndhK	NADH dehydrogenase
	psaA, psaB, psaC, psaI, psaJ, psaM	Photosystem I
	psbA, psbB, psbC, psbD, psbE, psbF, psbH, psbI, psbJ, psbK, psbL, psbM, psbN, psbT, psbZ	Photosystem II
	rpl2, rpl14, rpl16, rpl20, rpl21, rpl22, rpl23, rpl32, rpl33, rpl36	Large ribosomal protein units
	rps2, rps3, rps4, rps7, rps8, rps11, rps12, rps14, rps15, rps18, rps19	Small ribosomal protein units
	rrn4.5, rrn5, rrn16, rrn23	Ribosomal RNAs
	rpoA, rpoB, rpoC1, rpoC2	RNA polymerase
	rbcL	Rubisco large subunit
	cysA, cysT	Sulphate ABC transporter subunit
	trnA-UGC, trnC-GCA, trnD-GUC, trnE-UUC, trnF-GAA, trnfM-CAU, trnG-GCC, trnG-UCC, trnH-GUG, trnI-CAU, trnI-GAU, trnK-UUU, trnL-CAA, trnL-UAA, trnM-CAU, trnN-GUU, trnP-UGG, trnQ-UUG, trnR-ACG, trnR-CCG, trnR-UCU, trnS-GCU, trnS-UGA, trnT-GGU, trnT-UGU, trnV-GAC, trnV-UAC, trnW-CCA, trnY-GUA	Transfer RNAs
	infA	Translational initiation factor
**mtDNA**	atp1, atp4, atp6, atp8, atp9	ATP synthase
	ccmB, ccmC, ccmFC, ccmFN	Cytochrome c biogenesis
	cox1, cox2, cox3	Cytochrome c oxidase
	cob	Cytochrome c reductase
	rpl2, rpl5, rpl6	Large ribosomal protein units
	nad1, nad2, nad3, nad4, nad4L, nad5, nad6, nad9	NADH dehydrogenase
	rrn5, rrn18, rrn26	Ribosomal RNAs
	rps1, rps2, rps3, rps4, rps7, rps8, rps10, rps11, rps12, rps13, rps14, rps19	Small ribosomal protein units
	sdh3, sdh4	Succinate dehydrogenase
	trnA-UGC, trnC-GCA, trnC-UCC, trnD-GUC, trnE-UUC, trnF-GAA, trnG-GCC, trnG-UCC, trnH-GUG, trnI-CAU, trnK-UUU, trnL-CAA, trnL-UAA, trnL-UAG, trnM-CAU, trnMf-CAU, trnN-GUU, trnP-UGG, trnQ-UUG, trnR-ACG, trnR-UCU, trnS-GCU, trnS-UGA, trnV-UAC, trnW-CCA, trnY-GUA	Transfer RNAs
	tatC	Twin arginine subunit c

**Figure 2:**
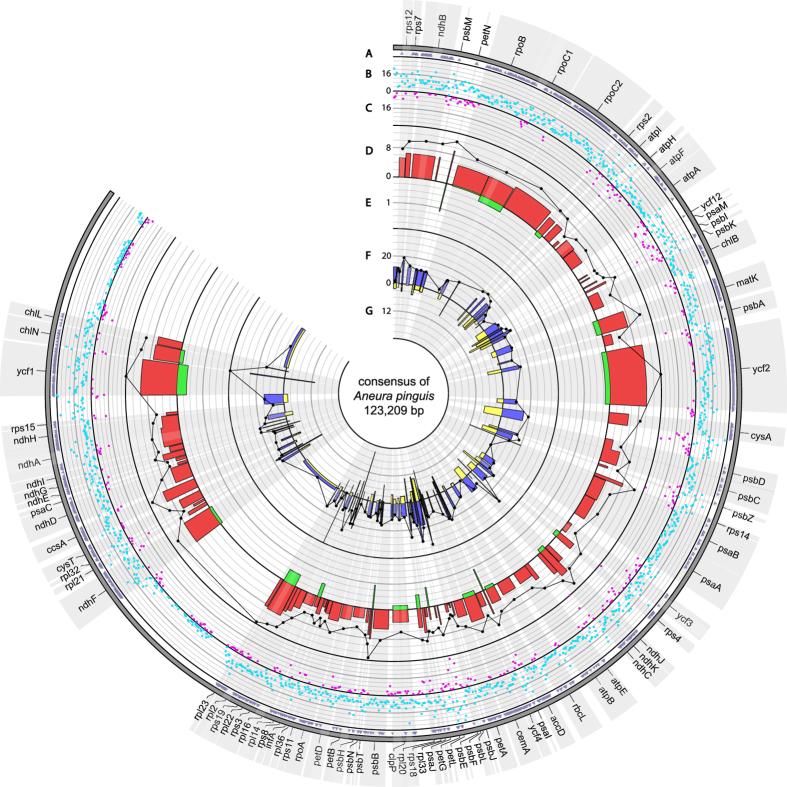
Circos graph representing SNP and indel variation among plastomes of six cryptic species of *A. pinguis*. Track A shows nonsynonymous SNP occurence within genes. Track B and C represent identified SNP (light blue spots) and indel (purple spots) per 100 bp, respectively. Track D represents percent of SNPs per CDS length while track E represents percent of indels per CDS length. Black plot represents π value (maximum value = 0.06) for each CDS. Track F represents percent of SNPs per noncoding region length while track G represents percent of indels per noncoding region length. Black plot represents π value (maximum value = 0.2) for each noncoding region.

A total of 182 regions were identified in the chloroplast genomes of the analysed cryptic species: 86 CDSs, 12 introns and 84 intergenic spacers. 10,169 SNPs and 1,537 indels were found across whole plastome of *Aneura pinguis*. Among the 5,455 SNPs identified in coding sequences, 2,232 are nonsynonymous and 3,223 are synonymous. Also 54 indels were found inside coding sequences (Fig. 2). A mean value of π for plastome was 0.05111.

In noncoding regions longer than 100 bp (to eliminate bias) the π value was the highest in *ycf*1*-chl*N spacer (0.230) (Supplementary Table S4). In this relatively short fragment (142 bp) 36 SNPs and 19 indels occured. The second most polymorphic spacer (π = 0.149), between *pet*L and *pet*G genes, contained 57 SNPs and 21 indels while 194 bp long. The *rpl*14*-rpl*16 spacer had slightly lower diversity (π = 0.131), however in this, 122 bp long spacer, only 15 SNPs and 9 indels occured.

Among introns the highest diversity (π = 0.073) was present in intron 1 of *ndh*A gene (800 bp) with 104 SNPs and 26 insertions and deletions (Supplementary Table S4). *ndh*B intron 1 showed a similar level of diversity (π = 0.068), although it was much shorter (624 bp). This region contained 77 SNPs and 3 indels. The third most polymorphic intron (π = 0.061) was *clp*P intron 2. In this, 423 bp long fragment, 52 SNPs and 10 indels occured.

In every coding region substitutions were detected. The nucleotide diversity varied from 0.014 to 0.062 (Supplementary Table S2). The most variable plastid genes were *ycf*1 and *ycf*2, coding proteins of unknown function. The *ycf*1 seems to be the most variable in the plastid genome and is repeatedly used in phylogenetic studies^53^ as well as DNA barcode^54^. Recently *ycf*2 gene is also recommended for this type of analysis showing a promising level of variation^55–57^. In those genes respectively 389 and 671 SNPs were found including 255 and 442 nonsynonymous substitutions. For comparison, 29 nonsynonymous substitutions have been found in *ycf*1 of genus *Pulsatilla* ^55^, but only 3 nonsynonymous substitutions have occurred in this gene of *Arabis alpina* ^58^. Similarly, few nonsynonymous substitutions have been found in the *ycf*2, for instance: three in *Tortula ruralis* ^59^ or only one in *Tetraplodon fuegianus* ^60^. In comparison, a variability of *ycf*1 and *ycf* 2 genes in *Aneura pinguis* is huge. The other genes of this group (*ycf*4, *ycf*3 and *ycf*12) appeared to be much less polymorphic. A high level of nonsynonymous SNPs has also been identified in genes such as: *rpl*32 (11 SNPs, 7 nonsynonymous), *ndh*G (47 SNPs, 27 nonsynonymous), *mat*K (143 SNPs, 82 nonsynonymous), *psb*K (14 SNPs, 8 nonsynonymous), *rpl*20 (27 SNPs, 15 nonsynonymous), *rpl*33 (9 SNPs, 5 nonsynonymous). Analysis of nonsynonymous substitutions occurrence showed a group of 4 genes, which were identified as highly conserved: *psa*M, *psb*I, *psb*L, *psb*F. In their coding sequences nonsynonymous substitutions were not identified. Also other genes of *psa* and *psb* family showed little polymorphism, probably due to the importance of its function, which is encoding subunits of photosystem I and II^52^.

### Structure and polymorphism of mitochondrial genome

The mitochondrial genomes of six cryptic species of *Aneura pinguis* length range from 164,047 to 167,033 bp (Fig. 1B), which is slightly shorter than the closest related species with known mitogenome structure – *Pleurozia purpurea* (168,526 bp)^18^. The two other known mitochondrial genomes of the liverwort species, *Treubia lacunosa* and *Marchantia polymorpha*, differ more and are composed of 151,983 and 186,609 bp, respectively. Overall GC content of *Aneura pinguis* is 47.4%, which is similar to other liverworts (42–45%)^15, 48^.

68 unique genes have been identified in *Aneura pinguis* mitogenome, including 40 protein coding genes, 3 ribosomal RNAs and 25 transfer RNAs (Table 3). Nine genes such as: *atp*9, *nad*2, *nad*3, *nad*4, *nad*5, *rpl*2, *rps*14, *rrn*18 and *trn*S-GCU contain one intron, four genes: *atp*1, *cox*2, *cox*3, *nad*4L contain two introns, *cob* contains 3 introns and *cox*1 contains 9 introns. Gene order is identical to the three aforementioned liverworts mitogenomes. However one exception has been observed. The *nad*7 gene was not identified in the obtained mitochondrial genome. The earlier analyses conducted on bryophytes have shown that *nad*7 in hornworts and in most liverworts is missing or frequently occurs as partial pseudogene with degenerated structure^17, 61, 62^ in contrast to mosses, in which apparently this gene is functional^63^. The only liverwort species with non-pseudogenised *nad*7 are *Haplomitrium* ^62^ and *Treubia* ^48^, which form a common clade, Haplomitriopsida, regarded as a sister to the rest of the liverworts (Marchantiopsida and Jungermanniopsida)^64^ having pseudogene *nad*7. The studied species *Aneura pinguis* (Jungermanniopsida) has a nonfunctional mitochondrial copy of *nad*7, which is in line with the above division of Marchantiophyta. Pseudogenization of this gene in *Aneura pinguis* seems to rely on the loss of exon 1 occurring in *nad*7 gene of *Treubia lacunosa* and in pseudogene of *Pleurozia purpurea*. Exons 2 and 3 are present and very similar to the exons 2 and 3 of *Treubia lacunosa*, to 80% and 94% respectively. The biggest changes occur in intron 2. This segment is 162 bp shorter than intron 2 of *Treubia lacunosa* and similar to it only to about 40%, whereas intron 1 of *Aneura pinguis* has 80% paired identity with the same segment in *Pleurozia purpurea*.

**Figure 3:**
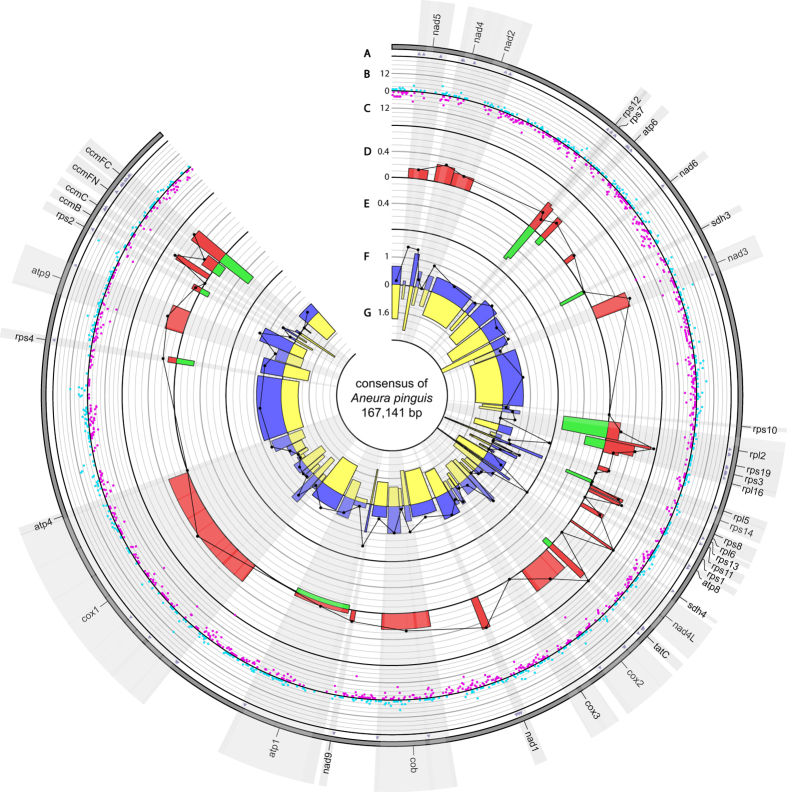
Circos graph representing SNP and indel variation among mitogenomes of six cryptic species of *A. pinguis*. Track A shows nonsynonymous SNP occurence within genes. Track B and C represent identified SNP (light blue spots) and indel (purple spots) per 100 bp, respectively. Track D represents percent of SNPs per CDS length while track E represents percent of indels per CDS length. Black plot represents π value (maximum value = 0.004) for each CDS. Track F represents percent of SNPs per noncoding region length while track G represents percent of indels per noncoding region length. Black plot represents π value (maximum value = 0.01) for each noncoding region.

103 regions have been identified in the mitochondrial genome of *Aneura pinguis*: 40 CDSs, 27 introns and 36 intergenic spacers. 953 SNPs and 1,940 indels have been identified in total. Among the 84 SNPs identified in coding sequences, 72 are nonsynonymous and 12 are synonymous. 43 indels were also found inside coding sequences (Fig. 3). A mean value of π for mitogenome was 0.00233.

The nucleotide diversity value among noncoding regions longer than 100 bp was the greatest in *sdh*4*-nad*4L spacer (π = 0.012407) (Supplementary Table S5). This spacer contained 5 SNPs and 14 indels while 412 bp long. The second most polymorphic spacer (π = 0.009675) is adjacent to *rps* gene family. The 482 bp *rps*14*-rps*8 spacer comprised 9 SNPs and 15 indels. Another one of the most diverse regions is *nad*9*-atp*1 spacer (π = 0.007692) with 4 SNPs and 8 indels on 390 bp long section. A slightly lower polymorphism (π = 0.006709) was found in *nad*5*-nad*4 spacer. This 1,008 bp long region contained 12 SNPs and 26 indels.

The highest diversity among introns (π = 0.006938) was identified in the first intron of *nad*5 gene (Supplementary Table S5). This intron contained 11 SNPs and 5 indels. The second most polymorphic intron (π = 0.006395) was *cox*2 intron 1. In this, 1061 bp long section, 13 SNPs and 13 indels occured. The intron 1 and intron 3 of *cob* gene showed a similar level of polymorphism (π = 0.004842, π = 0.004828 respectively) and took place the third position among the most polymorphic introns.

The nucleotide diversity values in coding regions varied between 0 to 0.004087 (Supplementary Table S3). By far the most diverse mitogenome gene was *sdh*4 responsible for succinate dehydrogenase (π = 0.004087). In its coding sequence 2 SNPs were found and all of them were nonsynonymous substitutions. Similar level of polymorphism was detected in *tat*C gene coding sequence (π = 0.004082). In this region 5 SNPs were identified with 4 nonsynonymous SNPs. Whereas in other gene of *sdh* family – *sdh*3 no polymorphism was identified. The third most polymorphic coding sequence in mt genome was *rps*19 with 2 SNPs. High level of nucleotide diversity was also observed in *ccm*C gene, where all 4 SNPs were nonsynonymous. Other coding sequences of *Aneura pinguis* mitochondrial genome presented the π values below 0.003. A group of 8 CDSs contained neither SNPs nor indels. Those highly conserved coding sequences were: *atp*4, *ccm*B, *cox*3, *nad*4L, *rpl*6, *rps*1, *rps*10 and *rps*14. The last one was reported to be the most polymorphic as it revealed comparative mitogenomics between *Physcomitrella patens* and *Marchantia polymorpha* ^65^.

In comparison with the genome of the plastid, the mitochondrial genome is less variable. Generally, in the cp coding sequences of *Anuera* there are far more substitutions (5,455) than in mt sequences encode proteins (84). Furthermore, the mean value of nucleotide diversity per genome (cp: π = 0.05111; mt: π = 0.00233) also indicated that more permanent is mt genome. However, the total number of indels in both genomes is similar (cp: 1537; mt: 1526). The results are consistent with a common observation that mitochondrial genomes of bryophytes are stable, in contrast to mt chromosomes of angiosperms^66^. The HKA test evidenced that these two genomes of *Aneura* differ from one another in diversity level and that at least one of the mentioned genomes deviates from neutral expectation (χ^2^ = 5.381; p = 0.0204, p < 0.05). The dN/dS ratio, calculated for each protein coding sequence of mt and cp genome, indicated which genes are probably under positive selection within *A. pinguis* complex. The Z-test showed, which values of dN/dS ratios are significantly larger than 1. In plastome 31 genes were under positive selection. For 24 genes the dN/dS ratio was significantly higher than 1 and in 7 genes only nonsynonymous substitutions occurred (Supplementary Table S3). The dN/dS ratio is also associated with the strength of selection. Values over 1 indicate positive selection, whereas much higher values indicate stronger selection^67^. The dN/dS ratio values of six of the chloroplast genes: *psa*C, *chl*L, *psb*N, *psb*E, *clp*P and *psb*K range between 3.8 and 29 (Supplementary Table S2). All of them are directly or indirectly associated with photosynthesis.

Photosynthesis genes of *Arabis alpina* was also suggested to be under selection, but negative. In these protein coding sequences only synonymous substitutions were found^58^. Although mt genomes of bryophytes are quite conservative, in the case of mitogenome of *A. pinguis* the number of nonsynonymous substitutions in protein encoding genes was even 3–4 times greater than in the case of the families Orthotrichaceae^68^, or Grimmiaceae^69^. However, only four mitochondrial genes within studied *A. pinguis* complex display positive selection (Supplementary Table S3). Three of them play important role in the respiratory chain: *ccm*FC, *cox*1and *nad*1, while *rps*3 encodes small ribosomal protein unit.

We also conducted branch-site test for positive selection among mitochondrial and chloroplast genes of liverworts (species specified in Supplementary Table S1) using BUSTED algorithm^40^. The test, combining diversity and divergence data, indicated that 3 mitochondrial and 9 chloroplast genes were under positive selection within liverworts clade (Supplementary Tables S2 and S3). These results are consistent with aforementioned diversity analyses conducted among *A. pinguis* cryptic species supporting hypothesis that plastid genomes are more variable than mitochondrial genomes. Branch-site test depicted that on about 11% of chloroplast and 7% of mitochondrial genes of liverworts acts positive selection (Supplementary Tables S2 and S3).

### Phylogenomics relationships

**Figure 4:**
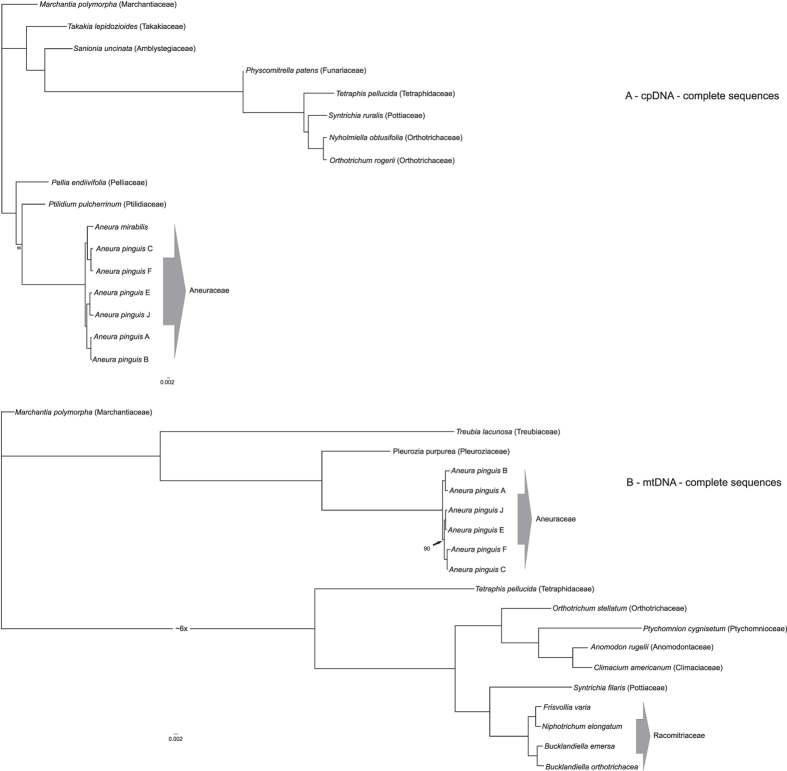
Phylogenetic relationships among *Aneura pinguis* cryptic species and other bryophytes based on complete sequences of plastomes (**A**) and complete sequences of mitogenomes (**B**). Bayesian inference phylogenetic trees of the 17 complete plastome (**A**) and 19 mitogenome (**B**) sequences of bryophytes. All resolved clades have maximum values of posterior probabilities. In case of the maximum likelihood analysis the values lower than 100% of bootstrap support are given at the nodes. The moss clade of the complete mitogenome analysis is scaled (six times shortened) for better representation of the phylogram.

**Figure 5:**
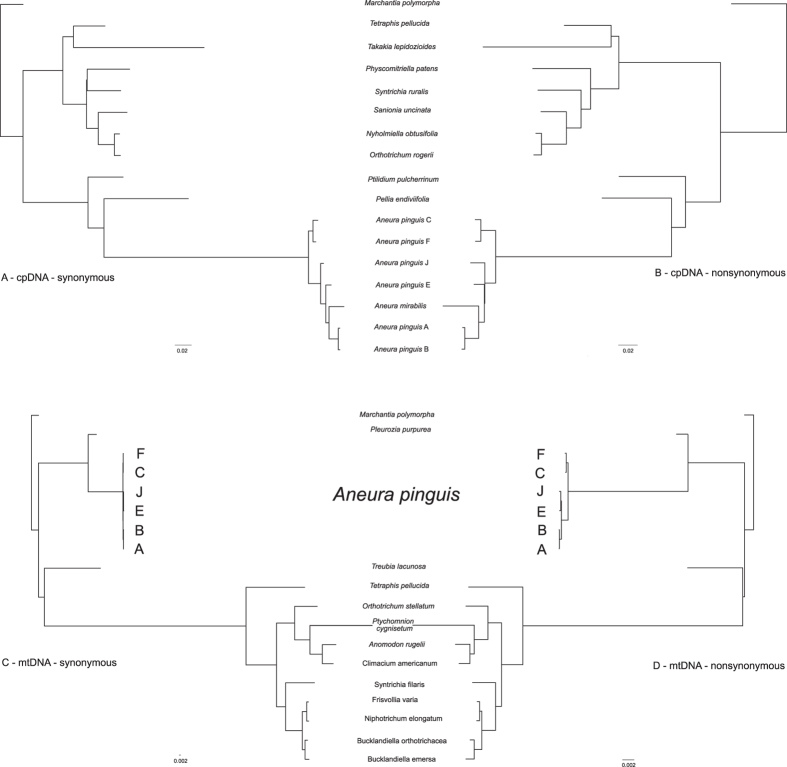
Phylogenetic relationships among *Aneura pinguis* cryptic species and other bryophytes based on partitioned plastome (**A** and **B**) and mitogenome (**C** and **D**) protein-coding datasets. Maximum likelihood analysis of protein-coding dataset was performed separately for synonymous (plastome - A, mitogenome - C) and nonsynonymous (plastome - B, mitogenome - D) sites. All resolved clades have maximum values of the maximum likelihood bootstrap support.

The phylogenetic trees based on chloroplast and mitochondrial genomes of 17 and 19 bryophytes based on Bayesian analysis are shown in Fig. 4. Both phylogenetic trees show that *Aneura pinguis* is differentiated and forms three distinct clades, very well being supported by Bayesian posterior probabilities (PP). The topology of trees generated by RAxML methods confirm the same relationships within the genus *Aneura* (Fig. 4). These analyses and many others studies indicated that *A. pinguis* is a paraphyletic taxon^49, 70, 71^ consisting of several evolutionary lineages corresponding to the previously detected cryptic species of *A. pinguis* ^3, 70^. The partitioned datasets based on synonymous and nonsynonymous substitutions revealed trees of identical topology as whole genome analyses (Fig. 5).

Relative-rate test revealed that both mitogenome and plastome of *A. pinguis* exhibits faster evolutionary rate than other tested liverworts, supporting our finding about extraordinary variation of the organellar genomes of this species within liverworts and formerly detected cryptic speciation of *A. pinguis* ^3, 70^. In comparison with moss species evolutionary rate of *A. pinguis* mitogenome did not differ significantly. However *A. pinguis* plastome evolutionary rate is higher than two of moss species (*Physcomitrella patens* and *Nyholmiella obtusifolia*) and lower than one moss species - *Takakia lepidozioides* (Table 2). These results are in accordance with the stability of the bryophyte mitogenome hypothesis and, on the other hand, proved higher plasticity of the bryophytes chloroplast genome.

*Aneura mirabilis* (previously belonging to the genus *Cryptothallus*) is a taxonomically uniform species in our studies. On the cpDNA trees, it is nested among the cryptic species of *A. pinguis* (Figs 4a and 5a,b). Moreover, molecular studies based both on coding (*rbc*L, *mat*K, *rpo*C1) and noncoding (*trn*L*-trn*F, *trn*H*-psb*A) barcode sequences also show that *A. mirabilis*, as well as *A. maxima*, are nested among the cryptic species of the *A. pinguis* complex (unpublished data). This indicates that the cryptic species of the *A. pinguis* complex do not derive directly from one common ancestor, but their evolutionary history is more complex.

## Conclusion

Due to next-generation sequencing one is able to present the first complete sequences of chloroplast and mitochondrial genomes of six cryptic species of *A. pinguis* complex. This method enabled the identification of highly variable sequences that could potentially be used as DNA barcode (e.g*. ycf*1, *ycf*2 or *sdh*4). The results also show that the aforementioned organellar genomes are extraordinarily variable, especially the chloroplast genome. About 36% of chloroplast genes in *A. pinguis* is under positive selection (based on Z-test). Surprisingly, taking into account the stability of mitogenome in bryophytes, on 10% of mitochondrial genes also acts directional selection. Moreover branch-site test showed that on about 11% of chloroplast and 7% of mitochondrial genes of studied liverworts acts positive selection. The above results indicate an advanced speciation of species. These findings confirm phylogenomic analyses, which divide *A. pinguis* into three distinct clades. The inclusion of the plastome sequence of *A. mirabilis* revealed paraphyly of *A. pinguis* as the former resolved as a sister to the cryptic species C and F. *A. pinguis* has a broad distribution and grows in various habitats, which might induce higher positive selection. Ecological processes are essential for the formation of new species, when barriers to gene flow (reproductive isolation) evolve between populations as a result of ecologically-based divergent selection. Although there are numerous studies providing evidence for the presence of ecological speciation, further lines of research are needed to explore the mechanisms underlying this process.

## Electronic supplementary material

Supplementary materials
