# Supplementary material for: The extraordinary variation of the organellar genomes of the Aneura pinguis revealed advanced cryptic speciation of the early land plants
Source: Sci Rep. 2017 Aug 29;7:9804. doi: 10.1038/s41598-017-10434-7 (PMC5575236; doi:10.1038/s41598-017-10434-7)
Supplement: Supplementary file 1 — Supplementary materials [file 41598_2017_10434_MOESM1_ESM.doc]

**SUPPLEMENTARY MATERIALS**

**The extraordinary variation of the organellar genomes of the Aneura pinguis revealed advanced cryptic speciation of the early land plants**

Kamil Myszczyński^1^*, Alina Bączkiewicz^2^, Katarzyna Buczkowska^2^, Monika Ślipiko^1^, Monika Szczecińska^1^, Jakub Sawicki^1^

^1^ Department of Botany and Nature Protection, University of Warmia and Mazury, Plac Łódzki 1, 10-727 Olsztyn, Poland

^2^ Department of Biology, Institute of Experimental Biology, Adam Mickiewicz University in Poznań, Umultowska 89, 61-614 Poznań, Poland

*Correspondence: kamil.myszczynski@gmail.com

| **Species** | **Taxonomy status** | **Mitochondrial genome length [bp]** | **Chloroplast genome length [bp]** | **GenBank accession number** | **Accession date** |
| --- | --- | --- | --- | --- | --- |
| *Aneura mirabilis* | Embryophyta; Marchantiophyta; Jungermanniopsida; Metzgeriidae; Metzgeriales; Aneuraceae; *Aneura* | - | 108,007 | NC010359 | 07.05.2009­­ |
| *Anomodon rugelii* | Embryophyta; Bryophyta; Bryophytina; Bryopsida; Bryidae; Hypnanae; Hypnales; Anomodontaceae; *Anomodon* | 104,239 | - | NC016121 | 21.10.2011­­ |
| *Bucklandiella orthotrichacea* | Embryophyta; Bryophyta; Bryophytina; Bryopsida; Dicranidae; Grimmiales; Grimmiaceae; *Bucklandiella* | 107,215 | - | NC026974 | 05.05.2015 |
| *Climacium americanum* | Embryophyta; Bryophyta; Bryophytina; Bryopsida; Bryidae; Hypnanae; Hypnales; Climaciaceae; *Climacium* | 105,048 | - | NC024515 | 03.06.2015 |
| *Codriophorus varius* | Embryophyta; Bryophyta; Bryophytina; Bryopsida; Dicranidae; Grimmiales; Grimmiaceae; *Codriophorus* | 106,358 | - | NC026891 | 22.04.2015 |
| *Marchantia polymorpha* | Embryophyta; Marchantiophyta; Marchantiopsida; Marchantiidae; Marchantiales; Marchantiaceae; *Marchantia* | 186,609 | 121,024 | NC001660  NC001319 | 09.04.2008  06.05.2009 |
| *Nyholmiella obtusifolia* | Embryophyta; Bryophyta; Bryophytina; Bryopsida; Bryidae; Bryanae; Orthotrichales; Orthotrichaceae; *Nyholmiella* | - | 122,895 | NC026979 | 05.05.2015 |
| *Orthotrichum rogeri* | Embryophyta; Bryophyta; Bryophytina; Bryopsida; Bryidae; Bryanae; Orthotrichales; Orthotrichaceae; *Orthotrichum* | - | 123,363 | NC026212 | 21.01.2015 |
| *Orthotrichum stellatum* | Embryophyta; Bryophyta; Bryophytina; Bryopsida; Bryidae; Bryanae; Orthotrichales; Orthotrichaceae; *Orthotrichum* | 104,131 | - | NC024522 | 03.06.2015 |
| *Pellia endiviifolia* | Embryophyta; Marchantiophyta; Jungermanniopsida; Pelliidae; Pelliales; Pelliaceae; *Apopellia* | - | 120,546 | NC019628 | 22.01.2013 |
| *Physcomitrella patens* | Embryophyta; Bryophyta; Bryophytina; Bryopsida; Funariidae; Funariales; Funariaceae; *Physcomitrella* | - | 122,890 | NC005087 | 26.03.2010 |
| *Pleurozia purpurea* | Embryophyta; Marchantiophyta; Jungermanniopsida; Metzgeriidae; Pleuroziales; Pleuroziaceae; *Pleurozia* | 168,526 | - | NC013444 | 17.10.2011 |
| *Ptilidium pulcherrimum* | Embryophyta; Marchantiophyta; Jungermanniopsida; Jungermanniidae; Ptilidiales; Ptilidiaceae; *Ptilidium* | - | 119,007 | NC015402 | 27.04.2011 |
| *Ptychomnion cygnisetum* | Embryophyta; Bryophyta; Bryophytina; Bryopsida; Bryidae; Hypnanae; Ptychomniales; Ptychomniaceae; *Ptychomnion* | 104,480 | - | NC024514 | 09.06.2015 |
| *Racomitrium elongatum* | Embryophyta; Bryophyta; Bryophytina; Bryopsida; Dicranidae; Grimmiales; Grimmiaceae; *Racomitrium* | 106,746 | - | NC026890 | 22.04.2015 |
| *Sanionia uncinata* | Embryophyta; Bryophyta; Bryophytina; Bryopsida; Bryidae; Hypnanae; Hypnales; Amblystegiaceae; *Sanionia* | - | 124,374 | NC025668 | 14.11.2014 |
| *Syntrichia filaris* | mbryophyta; Bryophyta; Bryophytina; Bryopsida; Dicranidae; Pottiales; Pottiaceae; *Syntrichia* | 106,343 | - | NC027515 | 14.07.2015 |
| *Syntrichia ruralis* | mbryophyta; Bryophyta; Bryophytina; Bryopsida; Dicranidae; Pottiales; Pottiaceae; *Syntrichia* | - | 122,630 | NC012052 | 16.03.2012 |
| *Takakia lepidozioides* | Embryophyta; Bryophyta; Takakiophytina; Takakiopsida; Takakiales; Takakiaceae; *Takakia* | - | 149,016 | NC028738 | 30.12.2016 |
| *Tetraphis pellucida* | Embryophyta; Bryophyta; Bryophytina; Tetraphidopsida; Tetraphidales; Tetraphidaceae; *Tetraphis* | 107,730 | 127,489 | NC024290  NC024291 | 26.01.2017  26.01.2017 |
| *Treubia lacunosa* | Embryophyta; Marchantiophyta; Haplomitriopsida; Treubiidae; Treubiales; Treubiaceae; *Treubia* | 151,983 | - | NC016122 | 21.10.2011 |

**Table S1. The specimens and taxonomy status of bryophytes used for phylogenomics reconstruction.**

| **Gene** | **Length** | **SNP** | **Indel** | **Nonsyn.** | **% PS** | **π** | **dN/dS** | **Z-test**  **p-value** | **BUSTED**  **p-value** |
| --- | --- | --- | --- | --- | --- | --- | --- | --- | --- |
| *ycf*1 | 3,276 | 389 | 12 | 255 | 12.240 | 0.062 | 1.051 | >0.05 | <0.041 |
| *ycf*2 | 5,607 | 671 | 10 | 442 | 12.145 | 0.061 | 0.505 | >0.05 | >0.05 |
| *rpl*23 | 276 | 29 | 0 | 14 | 10.507 | 0.058 | 0.533 | >0.05 | >0.05 |
| *psb*T | 108 | 11 | 1 | 3 | 11.111 | 0.057 | 0.062 | >0.05 | >0.05 |
| *ccs*A | 987 | 112 | 0 | 57 | 11.347 | 0.056 | 0.311 | >0.05 | <0.014 |
| *rpl*2 | 834 | 91 | 4 | 44 | 11.391 | 0.054 | 1.000 | >0.05 | >0.05 |
| *ndh*K | 696 | 74 | 1 | 33 | 10.776 | 0.053 | 1.190 | <0.048 | >0.05 |
| *ndh*F | 2,100 | 213 | 0 | 110 | 10.238 | 0.052 | 1.163 | <0.021 | >0.05 |
| *rps*19 | 279 | 26 | 2 | 10 | 9.3190 | 0.052 | 1.040 | >0.05 | >0.05 |
| *pet*N | 90 | 10 | 1 | 3 | 12.222 | 0.05 | - | <0.039 | >0.05 |
| *rps*14 | 303 | 31 | 0 | 10 | 10.231 | 0.05 | 1.724 | <0.042 | >0.05 |
| *mat*K | 1,521 | 143 | 3 | 82 | 9.599 | 0.049 | 0.350 | >0.05 | >0.05 |
| *chl*N | 1,404 | 128 | 2 | 40 | 9.259 | 0.046 | 1.469 | <0.048 | >0.05 |
| *cem*A | 1,320 | 117 | 0 | 60 | 9.015 | 0.044 | 0.289 | >0.05 | <0.005 |
| *ndh*B | 1,509 | 128 | 0 | 56 | 8.482 | 0.044 | 0.250 | >0.05 | >0.05 |
| *psa*I | 111 | 10 | 1 | 2 | 9.910 | 0.044 | 0.055 | >0.05 | >0.05 |
| *psb*M | 105 | 9 | 2 | 3 | 8.571 | 0.044 | 0.188 | >0.05 | >0.05 |
| *rpl*22 | 396 | 34 | 0 | 10 | 8.586 | 0.044 | 0.976 | >0.05 | >0.05 |
| *chl*L | 726 | 64 | 0 | 7 | 8.815 | 0.043 | 4.091 | <0.012 | >0.05 |
| *cys*T | 867 | 72 | 0 | 33 | 8.304 | 0.042 | 0.250 | >0.05 | >0.05 |
| *ndh*C | 405 | 36 | 0 | 16 | 8.889 | 0.042 | 1.625 | <0.042 | >0.05 |
| *rpo*C2 | 4,164 | 351 | 0 | 180 | 8.429 | 0.042 | 0.287 | >0.05 | <2.5 x 10^-4^ |
| *rps*7 | 468 | 40 | 0 | 9 | 8.547 | 0.042 | 0.080 | >0.05 | >0.05 |
| *psb*N | 132 | 12 | 0 | 3 | 9.091 | 0.041 | 4.000 | <0.041 | >0.05 |
| *psb*K | 168 | 14 | 0 | 8 | 8.333 | 0.039 | 3.000 | <0.022 | >0.05 |
| *rps*18 | 228 | 20 | 0 | 5 | 8.772 | 0.039 | 0.098 | >0.05 | >0.05 |
| *cys*A | 867 | 83 | 1 | 43 | 7.339 | 0.037 | 0.287 | >0.05 | >0.05 |
| *ndh*J | 477 | 35 | 5 | 10 | 7.337 | 0.037 | 2.056 | <0.014 | >0.05 |
| *rpl*20 | 375 | 27 | 0 | 15 | 7.733 | 0.037 | 0.778 | >0.05 | >0.05 |
| *rpo*B | 3,198 | 242 | 0 | 73 | 7.598 | 0.037 | 0.127 | >0.05 | >0.05 |
| *rpo*C1 | 2,082 | 159 | 2 | 57 | 7.877 | 0.037 | 0.141 | >0.05 | >0.05 |
| *psb*B | 1,518 | 110 | 1 | 17 | 7.246 | 0.036 | 0.056 | >0.05 | >0.05 |
| *rpo*A | 1,029 | 71 | 0 | 33 | 6.890 | 0.036 | 0.919 | >0.05 | >0.05 |
| *rps*2 | 834 | 51 | 0 | 13 | 7.222 | 0.036 | 0.073 | >0.05 | >0.05 |
| *ndh*D | 1,509 | 107 | 0 | 41 | 7.091 | 0.036 | 0.865 | >0.05 | >0.05 |
| *ndh*G | 579 | 47 | 0 | 27 | 8.117 | 0.036 | 0.889 | >0.05 | >0.05 |
| *atp*E | 417 | 29 | 0 | 5 | 6.954 | 0.035 | 2.111 | <0.021 | >0.05 |
| *atp*I | 747 | 53 | 1 | 17 | 7.095 | 0.035 | 0.141 | >0.05 | >0.05 |
| *pet*L | 96 | 7 | 0 | 5 | 7.292 | 0.035 | 0.687 | >0.05 | >0.05 |
| *rpl*21 | 351 | 27 | 0 | 12 | 7.692 | 0.035 | 0.211 | >0.05 | >0.05 |
| *rps*3 | 657 | 46 | 0 | 9 | 7.001 | 0.035 | 1.385 | <0.012 | >0.05 |
| *rps*4 | 609 | 43 | 0 | 15 | 7.225 | 0.035 | 2.846 | <0.037 | >0.05 |
| *ndh*A | 1,110 | 79 | 0 | 34 | 7.117 | 0.034 | 1.632 | <0.040 | >0.05 |
| *psb*C | 1,422 | 96 | 0 | 8 | 6.751 | 0.034 | 0.040 | >0.05 | >0.05 |
| *rps*12 | 69 | 5 | 0 | 2 | 7.246 | 0.033 | 0.235 | >0.05 | >0.05 |
| *pet*A | 963 | 63 | 0 | 26 | 6.542 | 0.032 | 0.225 | >0.05 | <0.02 |
| *rpl*36 | 114 | 6 | 0 | 2 | 5.263 | 0.032 | 0.339 | >0.05 | <2.6 x 10^-4^ |
| *rps*8 | 399 | 26 | 0 | 14 | 6.516 | 0.031 | 1.111 | <0.002 | >0.05 |
| *acc*D | 969 | 61 | 1 | 21 | 6.398 | 0.031 | 0.148 | >0.05 | >0.05 |
| *psb*H | 225 | 13 | 0 | 6 | 5.778 | 0.031 | 0.219 | >0.05 | >0.05 |
| *ndh*H | 1,152 | 75 | 1 | 19 | 6.510 | 0.03 | 0.213 | >0.05 | >0.05 |
| *ndh*I | 534 | 31 | 0 | 11 | 5.805 | 0.03 | 1.174 | <0.042 | >0.05 |
| *psa*J | 129 | 7 | 0 | 2 | 6.201 | 0.03 | 0.092 | >0.05 | >0.05 |
| *psb*J | 123 | 7 | 0 | 3 | 5.691 | 0.029 | - | <0.040 | >0.05 |
| *ycf*4 | 555 | 33 | 0 | 13 | 5.946 | 0.029 | 0.213 | >0.05 | >0.05 |
| *pet*D | 483 | 28 | 0 | 7 | 5.797 | 0.028 | 0.119 | >0.05 | <0.035 |
| *rpl*33 | 198 | 9 | 0 | 5 | 4.545 | 0.028 | 0.396 | >0.05 | <0.009 |
| *rpl*32 | 201 | 11 | 0 | 7 | 5.473 | 0.028 | 0.600 | >0.05 | >0.05 |
| *atp*A | 1,524 | 85 | 0 | 8 | 5.577 | 0.027 | 0.040 | >0.05 | >0.05 |
| *psb*I | 111 | 7 | 0 | 0 | 6.306 | 0.027 | - | <0.040 | >0.05 |
| *atp*B | 1,479 | 79 | 0 | 15 | 5.341 | 0.026 | 2.364 | <0.013 | >0.05 |
| *atp*F | 555 | 33 | 0 | 16 | 5.946 | 0.026 | 0.235 | >0.05 | >0.05 |
| *clp*P | 612 | 33 | 0 | 9 | 5.555 | 0.026 | 3.750 | <0.045 | >0.05 |
| *pet*G | 114 | 6 | 0 | 3 | 5.263 | 0.026 | 0.537 | >0.05 | >0.05 |
| *psa*B | 2,205 | 121 | 0 | 14 | 5.487 | 0.026 | 1.857 | <0.048 | >0.05 |
| *rbc*L | 1,428 | 79 | 1 | 9 | 5.532 | 0.026 | 0.041 | >0.05 | >0.05 |
| *chl*B | 1,542 | 78 | 0 | 16 | 5.058 | 0.025 | 0.068 | >0.05 | >0.05 |
| *rps*11 | 393 | 22 | 0 | 6 | 5.598 | 0.025 | 1.190 | <0.023 | >0.05 |
| *rps*15 | 270 | 14 | 0 | 5 | 5.185 | 0.025 | 0.793 | >0.05 | >0.05 |
| *psa*A | 2,253 | 117 | 0 | 12 | 5.193 | 0.024 | 2.556 | <0.046 | <1.6 x 10^-4^ |
| *psb*Z | 189 | 9 | 0 | 2 | 4.762 | 0.024 | 0.061 | >0.05 | >0.05 |
| *inf*A | 237 | 10 | 0 | 4 | 4.219 | 0.023 | 0.741 | >0.05 | >0.05 |
| *ndh*E | 303 | 15 | 0 | 5 | 4.950 | 0.023 | 0.680 | >0.05 | >0.05 |
| *pet*B | 648 | 31 | 0 | 6 | 4.784 | 0.023 | 0.077 | >0.05 | >0.05 |
| *psa*M | 99 | 5 | 2 | 0 | 5.050 | 0.023 | 0.000 | >0.05 | >0.05 |
| *rpl*14 | 369 | 18 | 0 | 7 | 5.420 | 0.023 | 1.786 | <0.042 | >0.05 |
| *rpl*16 | 414 | 21 | 0 | 7 | 5.072 | 0.023 | 1.190 | <0.007 | >0.05 |
| *psa*C | 246 | 12 | 0 | 2 | 4.878 | 0.022 | 29.000 | <0.023 | >0.05 |
| *ycf*12 | 102 | 5 | 0 | 1 | 4.902 | 0.022 | - | <0.040 | >0.05 |
| *psb*D | 1,062 | 45 | 0 | 7 | 4.237 | 0.02 | 0.057 | >0.05 | >0.05 |
| *psb*A | 1,062 | 47 | 0 | 2 | 4.426 | 0.019 | 0.013 | >0.05 | >0.05 |
| *atp*H | 246 | 10 | 0 | 4 | 4.065 | 0.018 | 0.256 | >0.05 | >0.05 |
| *psb*E | 252 | 11 | 0 | 1 | 4.365 | 0.018 | 3.833 | <0.042 | >0.05 |
| *psb*L | 117 | 4 | 0 | 0 | 3.419 | 0.016 | - | <0.40 | >0.05 |
| *ycf*3 | 393 | 13 | 0 | 4 | 3.308 | 0.015 | - | <0.013 | >0.05 |
| *psb*F | 120 | 3 | 0 | 0 | 2.500 | 0.014 | - | <0.04 | >0.05 |

**Table S2. SNP and indel variation within chloroplast genes of *A. pinguis*.**

Table represents SNP and indel occurrence within plastome genes including nonsynonymous SNPs. % PS - percent of polymorphic sites (percent of SNPs and indels per CDS length), π - nucleotide diversity, dN/dS - ratio of the number of nonsynonymous substitutions per nonsynonymous site to the number of synonymous substitutions per synonymous site, p-value - values of selection test of dN/dS ratio. s substitutions per synonymous site, p-value - values of selection test of dN/dS ratio. BUSTED p-value - p-values of branch-site statistical test. The shaded table fields indicates genes which selection test value was considered as significant (p-value < 0.05). Genes sorted by π value.

| **Gene** | **Length** | **SNP** | **Indel** | **Nonsyn.** | **% PS** | **π** | **dN/dS** | **Z-test**  **p-value** | **BUSTED**  **p-value** |
| --- | --- | --- | --- | --- | --- | --- | --- | --- | --- |
| *sdh*4 | 261 | 2 | 0 | 2 | 0.76628 | 0.00409 | 0.00 | >0.05 | >0.05 |
| *tat*C | 735 | 5 | 1 | 4 | 0.81633 | 0.00408 | 0.80 | >0.05 | >0.05 |
| *rps*19 | 282 | 2 | 0 | 0 | 0.70922 | 0.00378 | 0.00 | >0.05 | >0.05 |
| *ccm*C | 687 | 4 | 0 | 4 | 0.58224 | 0.00310 | - | >0.05 | >0.05 |
| *nad*3 | 357 | 2 | 0 | 2 | 0.56022 | 0.00299 | - | >0.05 | >0.05 |
| *ccm*FC | 1,446 | 8 | 8 | 7 | 1.10650 | 0.00285 | 4.00 | <0.024 | <1.5 x 10^-12^ |
| *rps*11 | 378 | 2 | 0 | 1 | 0.52910 | 0.00282 | 0.33 | >0.05 | >0.05 |
| *nad*1 | 987 | 5 | 0 | 5 | 0.50659 | 0.00250 | - | <0.024 | >0.05 |
| *rps*3 | 1,293 | 6 | 4 | 6 | 0.77339 | 0.00242 | - | <0.024 | <6 x 10^-10^ |
| *rps*13 | 363 | 2 | 0 | 1 | 0.55096 | 0.00239 | 0.00 | >0.05 | >0.05 |
| *cox*2 | 756 | 3 | 0 | 3 | 0.39682 | 0.00185 | - | >0.05 | >0.05 |
| *cox*1 | 1,569 | 5 | 0 | 4 | 0.31867 | 0.00170 | 2.00 | <0.024 | >0.05 |
| *atp*8 | 519 | 2 | 0 | 2 | 0.38536 | 0.00167 | - | >0.05 | >0.05 |
| *atp*6 | 777 | 3 | 1 | 3 | 0.51480 | 0.00155 | - | >0.05 | >0.05 |
| *atp*9 | 345 | 1 | 0 | 1 | 0.28985 | 0.00155 | - | >0.05 | >0.05 |
| *rps*12 | 639 | 3 | 4 | 3 | 1.09546 | 0.00147 | - | >0.05 | >0.05 |
| *nad*4 | 1,488 | 4 | 0 | 3 | 0.26882 | 0.00143 | 1.00 | >0.05 | >0.05 |
| *cob* | 1,215 | 3 | 0 | 2 | 0.24691 | 0.00137 | 0.50 | >0.05 | >0.05 |
| *rpl*2 | 1,584 | 3 | 11 | 3 | 0.88384 | 0.00107 | - | >0.05 | <7.5 x 10^-6^ |
| *rps*4 | 744 | 1 | 2 | 1 | 0.40323 | 0.00107 | - | >0.05 | >0.05 |
| *ccm*FN | 1,515 | 3 | 2 | 2 | 0.33003 | 0.00106 | 1.00 | >0.05 | >0.05 |
| *nad*2 | 1,470 | 3 | 0 | 2 | 0.20408 | 0.00095 | 0.50 | >0.05 | >0.05 |
| *rps*7 | 696 | 3 | 4 | 3 | 1.00575 | 0.00095 | - | >0.05 | >0.05 |
| *nad*6 | 600 | 1 | 0 | 1 | 0.16667 | 0.00089 | - | >0.05 | >0.05 |
| *rpl*5 | 600 | 1 | 0 | 1 | 0.16667 | 0.00089 | 0.00 | >0.05 | >0.05 |
| *nad*9 | 609 | 1 | 0 | 1 | 0.16420 | 0.00088 | - | >0.05 | >0.05 |
| *rpl*16 | 408 | 1 | 0 | 0 | 0.24510 | 0.00082 | 0.00 | >0.05 | >0.05 |
| *nad*5 | 2,010 | 3 | 0 | 3 | 0.14925 | 0.00070 | - | >0.05 | >0.05 |
| *rps*2 | 714 | 1 | 1 | 1 | 0.28011 | 0.00047 | 0.00 | >0.05 | >0.05 |
| *atp*1 | 1,542 | 1 | 1 | 1 | 0.12970 | 0.00035 | - | >0.05 | >0.05 |
| *rps*8 | 453 | 0 | 2 | 0 | 0.44150 | 0.00000 | - | >0.05 | >0.05 |
| *sdh*3 | 486 | 0 | 2 | 0 | 0.41152 | 0.00000 | - | >0.05 | >0.05 |
| *atp*4 | 615 | 0 | 0 | 0 | 0.00000 | 0.00000 | - | >0.05 | >0.05 |
| *ccm*B | 690 | 0 | 0 | 0 | 0.00000 | 0.00000 | - | >0.05 | >0.05 |
| *cox*3 | 798 | 0 | 0 | 0 | 0.00000 | 0.00000 | - | >0.05 | >0.05 |
| *nad*4L | 303 | 0 | 0 | 0 | 0.00000 | 0.00000 | - | >0.05 | >0.05 |
| *rpl*6 | 306 | 0 | 0 | 0 | 0.00000 | 0.00000 | - | >0.05 | >0.05 |
| *rps*1 | 771 | 0 | 0 | 0 | 0.00000 | 0.00000 | - | >0.05 | >0.05 |
| *rps*10 | 309 | 0 | 0 | 0 | 0.00000 | 0.00000 | - | >0.05 | >0.05 |
| *rps*14 | 300 | 0 | 0 | 0 | 0.00000 | 0.00000 | - | >0.05 | >0.05 |

**Table S3. SNP and indel variation within mitochondrial genes of *A. pinguis*.**

Table represents SNP and indel occurrence within mitochondrial genes including nonsynonymous SNPs. % PS - percent of polymorphic sites (percent of SNPs and indels per CDS length), π - nucleotide diversity, dN/dS - ratio of the number of nonsynonymous substitutions per nonsynonymous site to the number of synonymous substitutions per synonymous site, p-value - values of selection test of dN/dS ratio. BUSTED p-value - p-values of branch-site statistical test. The shaded table fields indicates genes which selection test value was considered as significant (p-value < 0.05). Genes sorted by π value.

| **Noncoding region** | **Length** | **SNP** | **Indel** | **% PS** | **π** |
| --- | --- | --- | --- | --- | --- |
| *ycf*1*-chl*N | 142 | 36 | 19 | 38.732 | 0.230 |
| *psb*L*-psb*F* | 53 | 16 | 7 | 43.396 | 0.167 |
| *ndh*J*-ndh*K* | 50 | 14 | 4 | 36.000 | 0.149 |
| *pet*L*-pet*G | 194 | 57 | 21 | 40.206 | 0.149 |
| *rpl*14*-rpl*16 | 122 | 15 | 9 | 19.672 | 0.131 |
| *rps*18*-rpl*20 | 142 | 30 | 15 | 31.690 | 0.128 |
| *acc*D*-psa*I | 224 | 43 | 18 | 27.232 | 0.106 |
| *ndh*E*-ndh*G* | 92 | 15 | 5 | 21.739 | 0.102 |
| *ndh*G*-ndh*I* | 81 | 15 | 1 | 19.753 | 0.102 |
| *psb*E*-pet*L | 742 | 135 | 73 | 28.032 | 0.101 |
| *rps*2*-atp*I | 269 | 43 | 19 | 23.048 | 0.099 |
| *psa*I*-ycf*4 | 259 | 51 | 15 | 25.483 | 0.099 |
| *cys*A*-psb*D | 1,670 | 264 | 130 | 23.593 | 0.098 |
| *rps*11*-rpl*36* | 61 | 14 | 13 | 44.262 | 0.098 |
| *inf*A*-rps*8* | 91 | 19 | 7 | 28.571 | 0.098 |
| *rps*7*-ndh*B | 238 | 41 | 8 | 20.588 | 0.095 |
| *rpo*C2*-rps*2 | 236 | 28 | 13 | 17.373 | 0.093 |
| *pet*G*-psa*J | 503 | 82 | 23 | 20.875 | 0.088 |
| *atp*I*-atp*H | 525 | 74 | 31 | 20.000 | 0.087 |
| *psb*Z*-rps*14 | 874 | 104 | 58 | 18.535 | 0.087 |
| *psb*I*-psb*K | 351 | 61 | 36 | 27.635 | 0.082 |
| *psb*B*-psb*T | 153 | 23 | 1 | 15.686 | 0.081 |
| *ndh*I*-ndh*A* | 77 | 12 | 0 | 15.584 | 0.081 |
| *rpl*21*-rpl*32 | 184 | 34 | 22 | 30.435 | 0.078 |
| *rpl*2*-rpl*23* | 77 | 11 | 2 | 16.883 | 0.076 |
| *ycf*4*-cem*A* | 82 | 12 | 2 | 17.073 | 0.075 |
| *psb*C*-psb*Z | 345 | 53 | 14 | 19.420 | 0.074 |
| *psa*J*-rpl*33 | 179 | 25 | 2 | 15.084 | 0.074 |
| *psb*T*-psb*N* | 76 | 11 | 6 | 22.368 | 0.073 |
| *ndh*A intron 1 | 800 | 104 | 26 | 16.250 | 0.073 |
| *psa*M*-psb*I | 555 | 62 | 35 | 17.477 | 0.072 |
| *psb*A*-ycf*2 | 628 | 82 | 37 | 18.949 | 0.072 |
| *rbc*L*-acc*D | 401 | 61 | 9 | 17.456 | 0.072 |
| *ndh*F*-rpl*21 | 377 | 56 | 20 | 20.159 | 0.072 |
| *ycf*3*-rps*4 | 380 | 56 | 32 | 23.158 | 0.071 |
| *chl*N*-chl*L* | 81 | 11 | 1 | 14.815 | 0.071 |
| *rpo*C1*-rpo*C2* | 93 | 12 | 8 | 21.505 | 0.070 |
| *rps*15*-ycf*1 | 1,781 | 252 | 32 | 15.946 | 0.069 |
| *ndh*B intron 1 | 624 | 77 | 3 | 12.820 | 0.068 |
| *atp*H*-atp*F | 291 | 39 | 18 | 19.588 | 0.068 |
| *pet*A*-psb*J | 174 | 22 | 4 | 14.942 | 0.067 |
| *psb*H*-pet*B | 210 | 29 | 3 | 15.238 | 0.067 |
| *cys*T*-ccs*A | 392 | 47 | 4 | 13.010 | 0.065 |
| *rpl*32*-cys*T | 150 | 21 | 4 | 16.667 | 0.063 |
| *ccs*A-*ndh*D | 317 | 27 | 13 | 12.618 | 0.063 |
| *rpl*16*-rps*3* | 596 | 8 | 3 | 16.176 | 0.062 |
| *chl*B*-mat*K | 1,142 | 139 | 23 | 14.186 | 0.061 |
| *clp*P intron 2 | 423 | 52 | 10 | 14.657 | 0.061 |
| *psb*J*-psb*L | 132 | 14 | 6 | 15.151 | 0.06 |
| *psb*M*-pet*N | 1,122 | 129 | 20 | 13.280 | 0.059 |
| *clp*P*-psb*B | 422 | 43 | 16 | 13.981 | 0.059 |
| *rps*19*-rpl*2* | 36 | 4 | 2 | 16.667 | 0.059 |
| *ndh*B*-psb*M | 402 | 43 | 17 | 14.925 | 0.058 |
| *pet*N*-rpo*B | 614 | 70 | 14 | 13.681 | 0.058 |
| *ycf*12*-psa*M | 262 | 29 | 21 | 19.084 | 0.058 |
| *pet*B*-pet*D | 153 | 17 | 7 | 15.686 | 0.058 |
| *psa*C*-ndh*E | 187 | 21 | 4 | 13.369 | 0.058 |
| *rps*12 intron 1 | 520 | 57 | 9 | 12.692 | 0.056 |
| *rpo*C1 intron 1 | 596 | 66 | 14 | 13.423 | 0.056 |
| *pet*D intron 1 | 581 | 62 | 18 | 13.769 | 0.056 |
| *atp*A*-ycf*12 | 1,296 | 132 | 57 | 14.583 | 0.055 |
| *psb*K*-chl*B | 462 | 49 | 25 | 16.017 | 0.055 |
| *ndh*C*-atp*E | 1,325 | 156 | 71 | 17.132 | 0.055 |
| *rps*14*-psa*B* | 50 | 10 | 6 | 32.000 | 0.054 |
| *ndh*D*-psa*C* | 87 | 10 | 2 | 13.793 | 0.054 |
| *rps*4*-ndh*J | 1,178 | 129 | 35 | 13.922 | 0.053 |
| *cem*A*-pet*A | 180 | 19 | 5 | 13.333 | 0.053 |
| *rpl*20-*clp*P | 987 | 95 | 29 | 12.563 | 0.052 |
| *rps*8*-rpl*14* | 57 | 5 | 1 | 10.526 | 0.052 |
| *rpl*16 intron 1 | 596 | 63 | 7 | 11.745 | 0.052 |
| *atp*B*-rbc*L | 626 | 60 | 8 | 10.863 | 0.049 |
| *ycf*2*-cys*A | 868 | 84 | 23 | 12.327 | 0.048 |
| *mat*K*-psb*A | 249 | 19 | 2 | 8.4337 | 0.046 |
| *ndh*K*-ndh*C* | 23 | 1 | 0 | 4.3478 | 0.046 |
| *rpl*2 intron 1 | 542 | 50 | 8 | 10.701 | 0.046 |
| *rps*12*-rps*7* | 51 | 5 | 0 | 9.8039 | 0.044 |
| *psa*A-*ycf*3 | 1,141 | 99 | 18 | 10.254 | 0.044 |
| *ycf*3 intron 1 | 689 | 56 | 17 | 10.595 | 0.043 |
| *rpl*33*-rps*18* | 80 | 8 | 0 | 10.000 | 0.043 |
| *pet*D*-rpo*A | 124 | 9 | 2 | 8.8710 | 0.043 |
| *clp*P intron 1 | 570 | 47 | 11 | 10.175 | 0.042 |
| *rpl*36*-inf*A* | 29 | 3 | 0 | 10.345 | 0.041 |
| *atp*F intron 1 | 623 | 49 | 7 | 8.989 | 0.04 |
| *pet*B intron 1 | 506 | 45 | 4 | 9.684 | 0.04 |
| *ndh*H*-rps*15* | 71 | 6 | 0 | 8.451 | 0.04 |
| *rpl*22*-rps*19* | 21 | 1 | 0 | 4.762 | 0.025 |
| *psb*N-*psb*H | 109 | 5 | 1 | 5.505 | 0.024 |
| *atp*F*-atp*A* | 44 | 2 | 1 | 6.818 | 0.02 |
| *rpo*A*-rps*11* | 28 | 1 | 1 | 7.143 | 0.02 |
| *rps*3*-rpl*22* | 7 | 3 | 0 | 42.86 | 0.02 |
| *chl*L*-rps*12 | 9,478 | 250 | 74 | 3.418 | 0.013 |
| *rpo*B*-rpo*C1* | 23 | 1 | 0 | 4.348 | 0.014 |
| *rpl*23-*ndh*F | 8,773 | 192 | 60 | 2.872 | 0.012 |
| *psb*F*-psb*E* | 18 | 0 | 1 | 5.555556 | 0 |
| *psa*B*-psa*A* | 26 | 0 | 0 | 0.000 | 0 |
| *atp*E*-atp*B* | 5 | 0 | 0 | 0.000 | 0 |

**Table S4. SNP and indel variation within chloroplast noncoding regions of *A. pinguis*.**

Table represents SNP and indel occurrence within plastome noncoding regions. % PS - percent of polymorphic sites (percent of SNPs and indels per CDS length), π - nucleotide diversity. Noncoding regions marked with asterisk were shorter than 100 bp. Regions sorted by π value.

| **Non-coding region** | **Length** | **SNP** | **Indel** | **% PS** | **π** |
| --- | --- | --- | --- | --- | --- |
| *sdh*4*-nad*4L | 412 | 5 | 14 | 4.6116 | 0.0124 |
| *rps*14*-rps*8 | 482 | 9 | 15 | 4.9792 | 0.0097 |
| *nad*9*-atp*1 | 390 | 4 | 8 | 3.0769 | 0.0077 |
| *nad*5 intron 1 | 624 | 11 | 5 | 0.9615 | 0.0069 |
| *nad*5*-nad*4 | 1,008 | 12 | 26 | 3.7698 | 0.0067 |
| *atp*9*-rps*2 | 2,973 | 35 | 25 | 2.0182 | 0.0064 |
| *cox*2 intron 1 | 1,061 | 13 | 13 | 2.4505 | 0.0064 |
| *rps*11*-rps*1 | 581 | 6 | 8 | 2.4096 | 0.0054 |
| *cob* intron 1 | 1,381 | 8 | 28 | 2.6068 | 0.0048 |
| *cob* intron 3 | 2,976 | 29 | 33 | 2.0833 | 0.0048 |
| *cox*1 intron 8 | 1,646 | 19 | 23 | 2.5516 | 0.0047 |
| *nad*2 intron 1 | 1,322 | 7 | 13 | 1.5129 | 0.0046 |
| *atp*6*-nad*6 | 3,989 | 32 | 67 | 2.4818 | 0.0042 |
| *cox*3-*nad*1 | 3,818 | 33 | 59 | 2.4096 | 0.0041 |
| *atp*1*-cox*1 | 6,759 | 41 | 126 | 2.4708 | 0.0040 |
| *atp*4*-rps*4 | 14,852 | 123 | 145 | 1.8718 | 0.0040 |
| *nad*3*-rps*10 | 13,442 | 100 | 210 | 2.3062 | 0.0039 |
| *cox*1 intron 1 | 2,741 | 18 | 23 | 1.4958 | 0.0036 |
| *rps*4*-atp*9 | 3,535 | 29 | 52 | 2.2914 | 0.0036 |
| *atp*8*-sdh*4 | 2,315 | 18 | 44 | 2.6782 | 0.0033 |
| *sdh*3*-nad*3 | 2,105 | 12 | 71 | 3.9430 | 0.0033 |
| *ccm*B*-ccm*C | 343 | 2 | 2 | 1.1662 | 0.0032 |
| *cox*3 intron 1 | 869 | 6 | 5 | 1.2658 | 0.0031 |
| *rps*14 intron 1 | 893 | 2 | 3 | 0.5599 | 0.0030 |
| *nad*4L intron 1 | 1,586 | 10 | 29 | 2.4590 | 0.0030 |
| *atp*1 intron 2 | 2,932 | 21 | 31 | 1.7735 | 0.0029 |
| *nad*1*-cob* | 6,187 | 28 | 120 | 2.3921 | 0.0028 |
| *rps*13*-rps*11 | 126 | 1 | 0 | 0.7936 | 0.0026 |
| *rpl*2 intron 1 | 756 | 5 | 10 | 1.9841 | 0.0026 |
| *nad*2*-rps*12 | 9,627 | 55 | 138 | 2.0048 | 0.0026 |
| *nad*6*-sdh*3 | 4,356 | 30 | 105 | 3.0992 | 0.0025 |
| *rps*7*-atp*6 | 1,058 | 5 | 14 | 1.7958 | 0.0024 |
| *cob-nad*9 | 3,673 | 15 | 50 | 1.7697 | 0.0024 |
| *atp*9 intron 1 | 2,821 | 12 | 25 | 1.3116 | 0.0024 |
| *ccm*FC*-nad*5 | 6,561 | 33 | 107 | 2.1338 | 0.0022 |
| *cox*3 intron 2 | 855 | 5 | 5 | 1.1696 | 0.0020 |
| *rpl*16*-rpl*5 | 1,963 | 10 | 28 | 1.9358 | 0.0020 |
| *rps*10*-rpl*2 | 790 | 2 | 9 | 1.3924 | 0.0018 |
| *nad*4 intron 1 | 1,586 | 5 | 9 | 1.6374 | 0.0014 |
| *cox*1 intron 6 | 1,052 | 3 | 12 | 1.4259 | 0.0014 |
| *cox*1 intron 9 | 1,048 | 4 | 4 | 0.7634 | 0.0013 |
| *cox*1 intron 7 | 1,042 | 3 | 6 | 0.8637 | 0.0012 |
| *rps*2*-ccm*B | 452 | 1 | 5 | 1.3274 | 0.0012 |
| *nad*4L intron 2 | 1,123 | 3 | 9 | 1.0686 | 0.0012 |
| *cox*2 intron 2 | 2,753 | 14 | 36 | 1.8162 | 0.0012 |
| *cox*1 intron 5 | 2,591 | 9 | 36 | 1.7368 | 0.0011 |
| *nad*3 intron 1 | 1,427 | 3 | 18 | 1.4716 | 0.0011 |
| *nad*4L*-tat*C | 528 | 1 | 5 | 1.1364 | 0.0010 |
| *cox*1 intron 2 | 2,583 | 6 | 15 | 0.8130 | 0.0010 |
| *atp*1 intron 1 | 3,314 | 6 | 7 | 0.3923 | 0.0008 |
| *rps*1*-atp*8 | 471 | 1 | 3 | 0.8493 | 0.0007 |
| *cox*2*-cox*3 | 807 | 1 | 7 | 0.9913 | 0.0007 |
| *cob* intron 2 | 884 | 1 | 9 | 1.1312 | 0.0006 |
| *ccm*C*-ccm*FN | 539 | 1 | 13 | 2.5974 | 0 |
| *ccm*FN*-ccm*FC | 204 | 0 | 1 | 0.4902 | 0 |
| *cox*1 intron 3 | 935 | 0 | 6 | 0.6417 | 0 |
| *cox*1 intron 4 | 1,063 | 1 | 3 | 0.3763 | 0 |
| *tat*C*-cox*2 | 453 | 0 | 4 | 0.8830 | 0 |
| *nad*4*-nad*2* | 26 | 0 | 0 | 0 | 0 |
| *rpl*2*-rps*19* | 3 | 0 | 0 | 0 | 0 |
| *rpl*6*-rps*13* | 3 | 0 | 0 | 0 | 0 |
| *rps*19*-rps*3* | 6 | 0 | 0 | 0 | 0 |
| *rps*8*-rpl*6* | 18 | 0 | 0 | 0 | 0 |

**Table S5. SNP and indel variation within mitochondrial noncoding regions of *A. pinguis*.**

Table represents SNP and indel occurrence within plastome noncoding regions. % PS - percent of polymorphic sites (percent of SNPs and indels per CDS length), π - nucleotide diversity. Noncoding regions marked with asterisk were shorter than 100 bp. Regions sorted by π value.
